# Genome-wide identification of the TIFY gene family in three cultivated *Gossypium* species and the expression of JAZ genes

**DOI:** 10.1038/srep42418

**Published:** 2017-02-10

**Authors:** Quan Sun, Guanghao Wang, Xiao Zhang, Xiangrui Zhang, Peng Qiao, Lu Long, Youlu Yuan, Yingfan Cai

**Affiliations:** 1 State Key Laboratory of Cotton Biology, Henan Key Laboratory of Plant Stress Biology, School of Life Sciences, School of Computer and Information Engineering, Henan University, Kaifeng 475004, China; 2 College of Bioinformation, Chongqing University of Posts and Telecommunications, Chongqing 400065, China; 3 State Key Laboratory of Cotton Biology, Cotton Institute of the Chinese Academy of Agricultural Sciences, Key Laboratory of Cotton Genetic Improvement, Ministry of Agriculture, Anyang, Henan 455000, China

## Abstract

TIFY proteins are plant-specific proteins containing TIFY, JAZ, PPD and ZML subfamilies. A total of 50, 54 and 28 members of the TIFY gene family in three cultivated cotton species—*Gossypium hirsutum, Gossypium barbadense* and *Gossypium arboretum—*were identified, respectively. The results of phylogenetic analysis showed that these TIFY genes were divided into eight clusters. The different clusters of gene family members often have similar gene structures, including the number of exons. The results of quantitative reverse transcription polymerase chain reaction (qRT-PCR) showed that different JAZ genes displayed distinct expression patterns in the leaves of upland cotton under treatment with Gibberellin (GA), methyl jasmonate (MeJA), Jasmonic acid (JA) and abscisic acid (ABA). Different groups of JAZ genes exhibited different expression patterns in cotton leaves infected with *Verticillium dahliae*. The results of the comparative analysis of TIFY genes in the three cultivated species will be useful for understanding the involvement of these genes in development and stress resistance in cotton.

TIFY family members, including JAZ subfamily proteins, play important roles in plant development, stress and hormone responses^1^,^2^,^3^,^4^. TIFY proteins are a class of plant-specific proteins with multiple functions and include the TIFY, Jasmonate ZIM-domain (JAZ), (PPD) and Zinc-finger protein expressed in Inflorescence Meristem (ZIM)-like protein (ZML) subfamilies^2^,^4^. TIFY proteins are key proteins in terrestrial plants. This family possesses a conserved zinc-finger protein domain and is expressed in the inflorescence meristem, characterized by an approximately 36-amino acid peptide pattern of TIF [F/Y]XG^1^,^2^. Previous studies have shown that the TIFY family contains four subfamily members (TIFY, ZML, JAZ and PPD). ZML protein has a specific TIFY domain similar to TLS[F/Y] XG. The zinc-finger proteins that are primarily expressed in the inflorescence meristem, namely, the (ZIM)-like (ZML) subfamily, include the ZIM and ZML proteins, which contain the CCT domain and C2C2-GATA zinc-finger domain. The JAZ subfamily contains the TIFY domain and the JA-associated (Jas, also named CCT_2) functional domain^1^,^3^,^4^,^5^. The Jas domain has a special consensus motif SLX2FX2KRX2RX5PY^4^,^5^,^6^. PEAPOD (PPD) subfamily proteins often contain a unique N-terminal PPD domain and an abnormal Jas motif lacking the conserved PY amino acids at the C-terminus^4^,^5^. Jasmonates (JAs) are essential phytohormones regulating plant development and responses to environment. In Jasmonic acid (JA) signalling, JAZ proteins regulate the expression of genes downstream of JA by binding to the transcription factor MYC2.

Previous studies have shown that ZML is involved in the regulation of diverse aspects of plant development and defence. In *Arabidopsis,* AtZIM (TIFY1) caused the elongation of hypocotyls and petioles^7^, ZML2 and ZML1 are involved in the cry1-mediated photoprotective response^8^, and ZmZML2 and ZmMYB11 form the MYB/ZML complex, involved in wound-induced lignification^9^.

In Arabidopsis, the PPD protein interacts with STERILE APETALA (SAP)^10^ and KIX8/9^11^ and plays a role in the regulation of tissue growth, modulation of lamina size, and limitation of the curvature of the leaf blade^12^,^13^. AtPPD4 may also have a function in defence against geminiviruses^12^.

JAZ protein is a key protein in the plant-specific JA signalling pathway. The JA content is relatively low in plants that are growing normally. When a plant is exposed to external stress or during development, the expression level of JA-isoleucine (JA-Ile) is often elevated. JAZ proteins bind to coronatine-insensitive 1 (COI1) through Skp1/Cullin1/F-box protein COI1 (SCFcoi1) complex-mediated ubiquitination and regulate ubiquitin-26S proteasome degradation. JA also induces the synthesis of JAZ proteins to inhibit the activity of transcription factors^3^,^14^,^15^,^16^,^17^,^18^.

In plants, JAZ proteins play important roles in stress responses, disease resistance, and plant development. In rice (*Oryza sativa*), overexpressing OsTIFY11a (OsJAZ9) increases tolerance to salt and drought and produces larger-sized grains^19^. In *Arabidopsis thaliana*, plants overexpressing AtJAZ10 are insensitive to JA. However, AtJAZ10 overexpression attenuates stress injury-induced growth inhibition. In *Arabidopsis thaliana*, the overexpression of GsJAZ2 (*Glycine soja*) results in an enhanced tolerance to salt and alkali stresses^20^. In native tobacco (*Nicotiana attenuata*), the overexpression of NaJAZd and NaJAZh inhibits the shedding of flower buds and promotes the synthesis of nicotine, respectively^21^,^22^,^23^. In cotton (*Gossypium hirsutum*), GhJAZ1 plays an important role in maintaining the balance between defence responses against Verticillium wilt and cotton growth^24^. In addition, studies have shown that JAZ genes play an important role in disease biotic and abiotic stress and hormonal signalling pathways^25^,^26^,^27^,^28^,^29^,^30^,^31^,^32^,^33^.

Recently, 28 TIFF gene family encoding proteins were identified in the wild cotton species *G. raimondii*^34^, an ancestor of cultivated tetraploid cotton species, which may be the origin of the D genome. The 28 TIFY gene sequences encoding TIFY proteins include 15 JAZ, 8 ZML, 3 PPD and 2 TIFY. Six genes (GrJAZ01, GrJAZ04, GrJAZ08, GrJAZ09, GrTIFY2, and GrZML6) showed altered expression levels during all stages of fibre growth. In a previous study of cotton transcriptomes, we showed that the cotton GhJAZ10 gene is induced through *Verticillium dahliae* and responds to Verticillium wilt. Until recently, the TIFY family was not systematically studied in cultivated cotton. Obtaining insights into the involvement of TIFY genes in evolution will aid in our understanding of cotton resistance to stresses. The completion of the genome sequences of cotton cultivars provides a basis for the genomic analysis of the entire TIFF gene family. In the present study, we systematically identified the TIFY family proteins using the cotton genome database^35^,^36^,^37^. Using fluorescence-based quantitative reverse transcription polymerase chain reaction (qRT-PCR) assays, we further analysed JAZ protein expression patterns in various tissues of cotton and explored changes in JAZ transcript levels in cotton leaves after treatment with various hormones and infection with *Verticillium dahliae*. We successfully discovered the expression patterns of the JAZ proteins in cotton plants in response to hormone treatment and *Verticillium dahliae* infection. The present study lays the foundation for the further identification of common signalling pathways controlled through JA and other hormones and the mechanisms underlying stress and disease resistance in cotton.

## Results

### Identification of members of the TIFY family in cotton

To identify TIFY-related proteins in cotton genomes, the HMMER profile was implemented to identify the genomes of *Gossypium hirsutum, Gossypium barbadense* and *Gossypium arboreum* databases. The results showed that a total of 28 *Ga*TIFY, 54 *Gb*TIFY and 50 *Gh*TIFY genes were characterized from cotton databases. These genes were further analysed using SMART and PFAM to verify that their coding amino acid sequences have the TIFY, Jas, ZIM and PPD domains. The encoded proteins comprised between 60 (*Gh*TIFY4) and 1114 (*Ga*JAZ07) amino acid residues, and the molecular weight was distributed from 7.10 (*Gh*TIFY4) to 122.0 (*Ga*JAZ07) kDa (Supplemental Table 1).

The chromosomal distributions of the TIFY gene family of *Gossypium hirsutum* and *Gossypium arboreum* were analysed. The results revealed 38 *GhTIFY* genes in the chromosome location information. Some chromosomes have several TIFY gene family members: six TIFY genes were located on No. Dt_chr9, four TIFY genes were detected on No. At_chr9, and three genes each were detected on No. At_chr8, Dt_chr2 and Dt_chr5 (Supplemental Fig. 1). For *Gossypium arboreum,* all predicted TIFY genes were detected in the chromosome localization information. Four TIFY genes were each observed on chr10, chr8 and chr6, and two TIFY genes were each detected on chr1, chr2, chr3, chr5, chr9 and chr12 (Supplemental Fig. 1). For *Gossypium barbadense,* the information on the location of the predicted TIFY gene chromosomes was not observed, as genome assembly was not complete.

### Phylogenetic analysis of the TIFY family genes

To gain further insights into the phylogenetic relationship among PPD, JAZ and ZML genes, the identified upland cotton^34^, grape^38^, Arabidopsis^2^, and rice^19^ TIFY protein sequences were used to conduct a multiple sequence alignment and construct a phylogenetic tree. The results showed that these proteins were divided into 8 main clusters labelled with JAZI-JAZVI PPD and ZIM and ZML (Supplemental Fig. 2). In the 8 clusters, multiple homologous genes of different species were observed in each cluster, except the JAZ1 subfamily. In the JAZ1 cluster, only *Oryza sativa* TIFY genes were observed, suggesting that the rice-specific gained genes were observed in this clade.

In the phylogenetic tree, these genes, including *GhJAZ1/GhJAZ2, GhJAZ3/GhJAZ4, GhJAZ6/GhJAZ24, GhJAZ7/GhJAZ13, GhJAZ8/GhJAZ14, GhJAZ9/GhJAZ17, GhJAZ18/GhJAZ19, GhJAZ20/GhJAZ21* and *GhJAZ22/GhJAZ23*, were clustered into a clade. The results via analysis of the sequence similarity of the coupled genes listed above showed similarities up to 99%, implying that these gene couples may originate from tetraploid homologous chromosomes.

**Figure 1:**
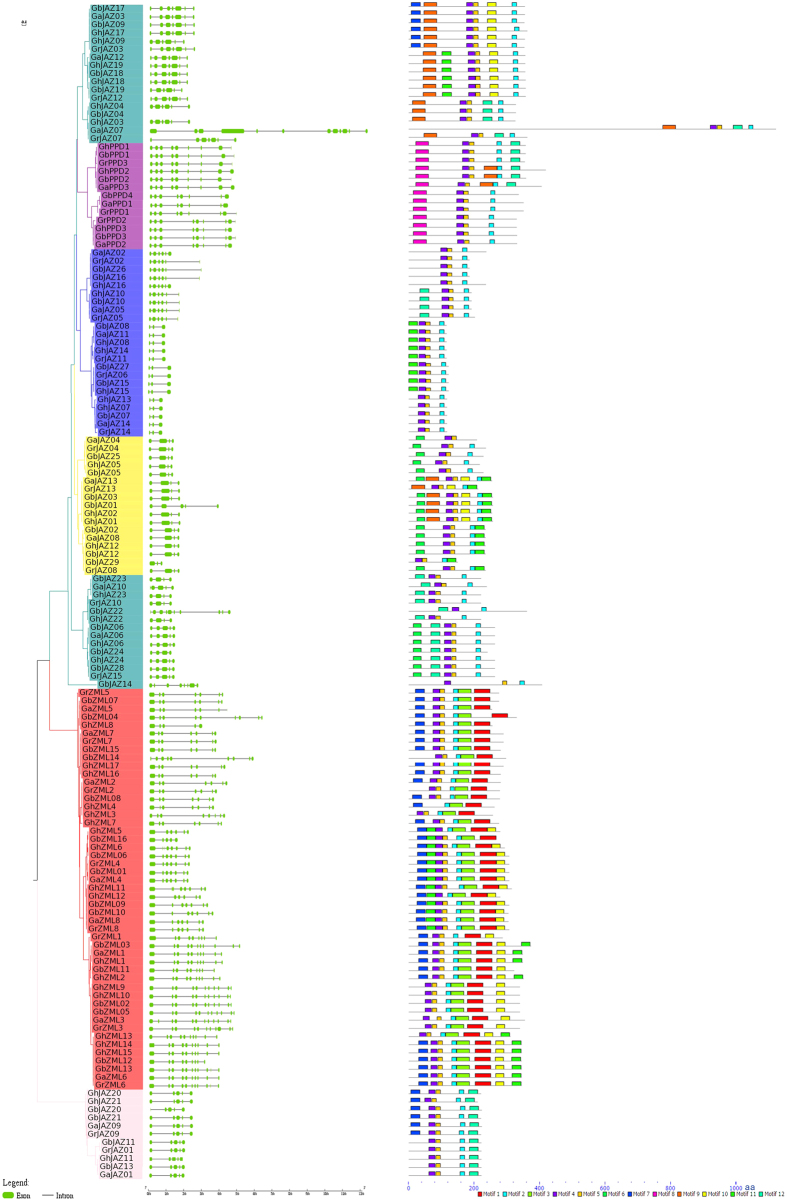
Phylogenetic analysis, gene structure and motif compositions of the TIFY gene family in cotton.

In addition, the genes encoding *Gossypium* TIFY gene family sequences were used for phylogenetic analysis. The results showed that these proteins were primarily divided into 7 clades (indicated with different background colours) (Fig. 1). As expected, the homologous genes in four cotton species often cluster together on one branch, e.g., GhZML11/GhZML12/GbZML09/GbZML10/GaZML8/GrZML8, often including two homologous genes in tetraploid cotton and one homologous gene in diploid cotton. The analysis of the characteristic motif also showed that the coding proteins on one branch often have similar motifs (Fig. 1).

### Evolution analysis of the TIFY family genes

**Figure 2:**
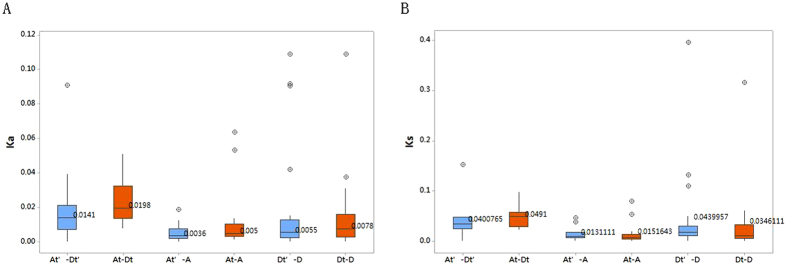
Comparison of Ka and Ks distributions in the A and D subgenomes. Orthologous or paralogs gene sets between the A genome, D genome and four subgenomes of *Gossypium hirsutum*(AtDt), *Gossypium barbadense*(At’Dt’).

To our knowledge, upland and sea-island cotton species including A and D subgenomes were likely derived from ‘A genome’ ancestors resembling *Gossypium herbaceum* (diploid) and ‘D genome’ species resembling *Gossypium raimondii* (diploid). The Ka/Ks values of the homologous genes from the same chromosomes (e.g., GhJAZ8/GhJAZ14 from the D genome 7/9) or from the A and D subgenomes (not including those positioned on the scaffolds of genes) were calculated, and the results showed that the Ka/Ks ratio values were <1, except for seven paired genes (Supplemental Table 2) (Fig. 2). The results showed 7 paired Ka/Ks gene ratios above 1, including Gb ZML03/GbZML11, GaJAZ3/GhJAZ9, GaPPD3/GhPPD2, GaZML5/GhZML8, GrJAZ05/GhJAZ10, GrZML1/GhZML2 and GrTIFY2/GhTIFY6. Among these paired genes, GaJAZ3/GhJAZ9, GaPPD3/GhPPD2, and GaZML5/GhZML8 included one *Gossypium arboretum* gene and one *Gossypium hirsutum* gene, and GrJAZ05/GhJAZ10, GrZML1/GhZML2, GrTIFY2/GhTIFY6 included one *Gossypium raimondii* gene and one *Gossypium hirsutum* gene. The Ka/Ks ratio values >1 suggest that these genes may have played a key role in the evolution of allotetraploid *Gossypium hirsutum* (upland cotton), as these genes were likely obtained from an ‘A genome’ ancestor resembling *Gossypium herbaceum* (diploid) and a ‘D genome’ species resembling *Gossypium raimondii* (diploid).

### Analysis of the TIFY gene structure

The exon/intron structure analysis showed that the same clades often have similar structures, including the number of exons/introns (Fig. 1). As shown in Fig. 1, among the 7 branches, from top to bottom, the genes on the first branch primarily include 6 or 7 exons, the genes on the second branch (purple) often have 8 or 9 exons, the blue branch genes have 3 or 6 exons, those on the yellow branch primarily have 4 exons, the fifth branch genes have 4 or 5 exons, the red branch genes have 7–11 exons and the genes on the bottom branch genes have 5 exons. The structure of the TIFY gene in *G. hirsutum* is closely associated with the topological structure of the evolutionary tree, and the gene structure is similar to that of members of the same branch. In G. arboreum, multiple genes in the same branch (such as GbJAZ17 GaJAZ03/GbJAZ09/GhJAZ17/GrJAZ03) have the same exon/intron number, and the length of the nucleotide is similar, but GhJAZ09, which is also in that branch, comparatively lacks the first exon, implying that in the evolution of *G. hirsutum*, this exon was lost. GhJAZ23, GbJAZ23 and GaJAZ10 are all in the same branch but do not have the first exon of GaJAZ10, indicating that from the evolution of *G. arboreum* (diploid Gossypium) to *G. hirsutum* and *G. barbadense* (tetraploid Gossypium), some of the exons were lost. GbJAZ01 has 5 exons, while other genes in the same branch only contain 4 exons, and their intron length is longer than that of the other genes, indicating that during evolution, *Gossypium barbadense* acquired a new exon.

The results of the analysis of the structure of the PPD and ZML protein domains showed that GbZML04 in sea-island cotton has two GATA domains, GbJAZ22 has two Jas domains, and GbTIFY4 and GbJAZ14 each have two TIFY domains.

### The cis-acting elements of JAZ genes

The promoter region often includes a variety of cis-acting elements and regulates gene time and space expression levels. In the present study, the upstream sequence of JAZ subfamily genes were used for cis-acting element prediction. The results showed that the JAZ genes contained various resistance- and hormone-related cis-acting elements (Supplemental Fig. 3). The hormone-related cis-acting elements primarily include the abscisic acid (ABA)-responsive elements ACGTATEERD1 and EBOXBNNAPA, the GA-responsive element WRKY710S and the salicylic acid (SA)-responsive elements GT1CONSENSUS and WBOXATNPR1. In addition, the JAZ genes contain a large number of stress resistance- and disease resistance-related elements, such as the salt-responsive element GT1GMSCAM4, defence- and stress-related element DOFCOREZM, pathogen elicitor-responsive element OSE2ROOTNODULE and mechanical injury-response element WBOXNTERF3. The JAZ genes also contained elements contributing to tissue-specific expression, including the leaf-specific element CACTFTPPCA1, stomatal guard cell-specific element TAAAGSTKST1, pollen-specific elements GTGANTG10 and POLLEN1LELAT52, root-specific element ROOTMOTIFTAPOX1 and seed-specific element SEF4MOTIFGM7S. In addition, JAZ genes contained other functional elements, such as light- and PAL-responsive elements.

### Expression patterns of the JAZ genes

Previous studies have shown that JAZ genes rapidly respond to JA and stress. The expression of JAZ genes will inhibit responses to JA signals in plants. The present study revealed that the JAZ genes exhibited a variety of distinct expression patterns after treating upland cotton seedlings with JA, MeJAZ, GA and ABA. The varying patterns of JAZ gene expression indicated that the JAZ genes responded to hormonal signals at the transcriptional level, thereby exerting similar or different functions. Most JAZ genes were rapidly upregulated and subsequently quickly downregulated after receiving exogenous JA or MeJA signals. Among the JAZ genes, JAZ8/14 displayed the most prominent changes in expression levels. The expression level of JAZ8/14 was upregulated 7.8-fold within 1 h of MeJA treatment and subsequently returned to a normal level at 6 h after MeJA treatment. However, certain JAZ genes, such as JAZ21, displayed a low degree of expression fluctuation after JA or MeJA treatment. In addition, the expression levels of a number of JAZ genes, such as JAZ6/24, JAZ16 and JAZ18/19, gradually increased over time following JA or MeJA treatment. In contrast, the expression levels of certain JAZ genes, such as JAZ15 and JAZ17, were downregulated after JA treatment. These findings demonstrate that different JAZ genes possess distinct functions.

**Figure 3:**
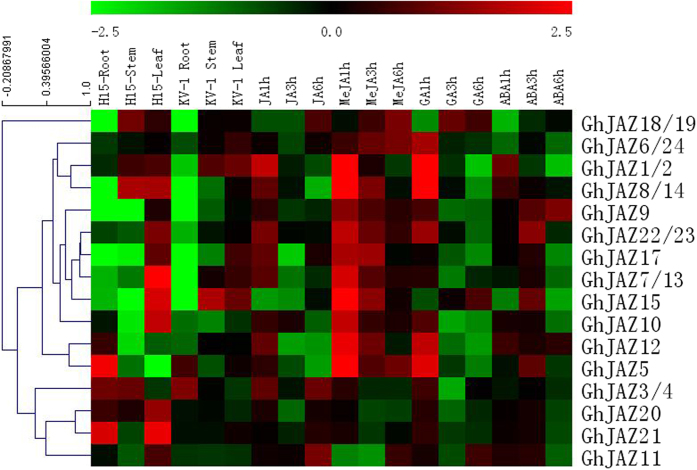
Expression profile of *JAZ* genes in cotton. The samples from left to right were Under normal growth state roots, stem, leaf of cotton varieties H15 and KV-1; H15 leaves treated by JA, MeJA, ABA, GA at 1 h, 3 h, 6 h. The bar showed gene expression value.

More than half of the JAZ genes quickly responded to GA signalling. The expression levels of JAZ1/2, JAZ5, JAZ6/24, JAZ8/14, JAZ9, JAZ10, JAZ12, JAZ16, JAZ18/19 and JAZ20 were upregulated shortly after GA treatment and subsequently decreased over time. ABA treatment induced limited changes in the expression levels of AZ3/4, JAZ5, JAZ7/13, JAZ12, JAZ20 and JAZ21. After ABA treatment, the expression of certain JAZ genes, such as JAZ1/2, was gradually downregulated over time. In contrast, the expression levels of certain JAZ genes (such as JAZ9) were rapidly downregulated after exposure to ABA and subsequently gradually returned to normal levels (Fig. 3).

In upland cotton, the transcript levels of the JAZ1/2, JAZ6/24, JAZ7/13, JAZ8/14, JAZ9, JAZ10, JAZ17, JAZ21 and JAZ22/23 genes were highest in the leaves. In contrast, the JAZ3/4 and JAZ5 genes showed the highest level of transcription in the roots, while the JAZ15 gene showed the highest transcription in the stems. The JAZ18/19 and JAZ12 genes were expressed at the lowest levels in the roots and, to a greater extent, in the stems and leaves. In addition, the expression levels of the JAZ18/19 and JAZ12 genes in stems were similar to those in the leaves. The JAZ11 and JAZ20 genes were transcribed at similar levels in the roots, stems and leaves.

**Figure 4:**
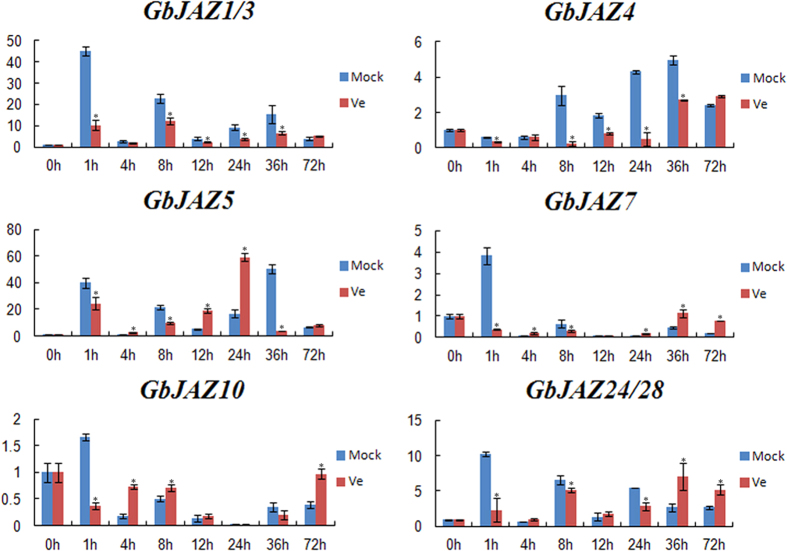
*JAZ* genes expression infected by *Verticillium dahliae* V991 in sea-island cotton.

After the infection of sea-island cotton with *Verticillium dahliae* of V991, the four groups of JAZ genes exhibited distinct expression patterns. The expression levels of GbJAZ1/3, GbJAZ24/28, GbJAZ7 and GbJAZ8 were downregulated at 1 h after infection compared to those in the Mock infection. The expression of JAZ1/3 and JAZ4 in the leaves showed a downward trend after *Verticillium dahliae* infection. The expression of JAZ5 was downregulated within the first 8 h after *Verticillium dahliae* infection and increased between 12 and 24 h, decreased again at 36 h and was eventually restored to the normal level. The expression of JAZ24/28 was downregulated in the first 24 h after *Verticillium dahliae* infection and downregulated again after 36 h. The expression of GbJAZ7 was slightly changed between 4 and 24 h; GbJAZ10 was upregulated at 4 and 72 h, and only a slight change was observed between 8–36 h. These results showed that various groups of JAZ genes displayed distinct expression patterns in the leaves of cotton plants (Fig. 4).

## Discussion

JAZ genes belong to the TIFY family and are members of a plant-specific gene family. The most primitive green algae do not contain TIFY genes. However, two TIFY genes have been identified in the earliest terrestrial plant moss, and the TIFY family of genes has been detected in all terrestrial plants^39^,^40^. Therefore, it is hypothesized that the TIFY gene family emerged during the evolution of plants from aquatic to terrestrial environments^41^.

Applying the genome sequence of the three cultivated Gossypium species, a total of 50, 54 and 28 TIFY genes were identified in *G. hirsutum, G. barbadense* and *G. arboretum*, respectively, in the present study. A total of 28 TIFY genes have been identified in *Gossypium raimondii*^34^. It is a typical polyploidization phenomenon that the number of TIFY genes in polyploid genomes (AADD) is almost double that in diploid genomes (AA or DD). However, 2 and 6 homology TIFY genes remain unidentified in *Gossypium hirsutum* and *Gossypium barbadense*, respectively, and the phylogenetic tree showed that the GbJAZ14 gene clustered independently with only one homologous gene; thus, the absent homologous genes may have been lost in evolution (Fig. 1). Because the GbJAZ14 gene may have no homologous genes in other cotton varieties, except *Gossypium barbadense*, GbJAZ14 may play important and unique roles in *Gossypium barbadense*. Changes in the intron-exon structure of the genes in the TIFY family play an important part in the evolution of this gene family^42^. The number and length of introns or exons are associated with the topological structure of phylogenetic tree in *G. raimondii*; in particular, a high degree of similarity was detected with respect to the structure and number of exons/introns between JAZ genes in the same cluster^34^, consistent with the results of the present study.

In all family members, the sequence showed obvious differences, and some of the sequences have conserved domains, such as Jas and ZIM. In addition, the similar gene exon/intron structures in the same clade of phylogenetic trees indicates that the introns in homologous genes display a conservative evolution tendency, consistent with the findings obtained in *Arabidopsis thaliana*^1^, rice^19^ and grape^38^. Comparison of the phylogenetic trees showed that the JAZ gene clusters were uniformly distributed in the phylogenetic trees, regardless of whether the JAZ genes originated from monocots or dicots. The results indicate that many plant-specific gene families exhibit considerable diversity in monocots and dicots. In addition, the clustering of most JAZ family genes included the selected plants, suggesting that the monocot and dicot JAZ genes may share a common ancestor.

The motif results showed that motif 3 was an NT domain. However, the NT domain is not required for interactions between JAZ proteins and MYC2, and other functions of the NT domain have been reported in plants. In rice, the proteins containing the NT domain respond quickly to JA stimulation^4^. Motif 8 in proteins GhJAZ7, GhJAZ8, GhJAZ13 and GhJAZ14 and the N-terminal 8–13 amino acids of protein GhJAZ15 contains the conserved “LXLXL” sequence. The LXLXL motif belongs to the EAR domain. The LXLXL motif is also present in AUX/IAA proteins and serves as a binding motif for the regulatory repressor TPLOSS. JAZ proteins are also transcriptional repressors, which interact with TPLOSS through similar mechanisms. The results of the present study showed that these upland cotton JAZ genes belong to the same clade, sharing a high degree of sequence similarity (up to 99%). It was difficult to design different qRT-PCR primers to distinguish the same clade among homologous genes. Therefore, the expression profile may include the transcription of two genes. In addition, certain JAZ genes, such as JAZ9 and JAZ17, displayed highly similar expression patterns, although the genes bore sufficient sequence differences and could be distinguished using quantitative PCR. JAZ20 and JAZ21 represented another example of such JAZ genes. Most JAZ genes were highly expressed in the leaves. The expression patterns of JAZ genes in upland cotton are similar to those in rice^19^, suggesting that these genes may play similar roles as their homologous genes.

In the present study, the sea-island cotton variety Xinhai 16 was infected with the highly virulent defoliating *Verticillium dahliae* strain V991 through the root tips and the expression patterns of various groups of JAZ genes in the leaves of the infected cotton plants were examined. The results showed that the JAZ genes responded to *Verticillium dahliae* infection and displayed various distinct expression patterns. Studies have reported that in upland cotton, GhJAZ1 responds to infection with *Verticillium dahliae*. GhJAZ1 binds to DELLA proteins, thereby regulating the balance between growth and defence^40^. In the present study, JAZ10 may be associated with the resistance of *V. dahliae* observed in a previous study^48^. These findings indicate that the JAZ genes of JA signalling pathways may play an important role in responses to *Verticillium dahliae* infection.

Promoters play a key role in the regulation of gene expression. In the present study, a 2000-base pair (bp) fragment upstream of the start codon of a gene was used for further analysis. A large number of functional elements were discovered, including the ABA-responsive elements ACGTATEERD1 and EBOXBNNAPA, the GA-responsive element WRKY710S, and the SA-responsive elements GT1CONSENSUS and WBOXATNPR1. In addition to the hormone response elements, a number of stress and disease resistance-related elements were identified. In *Arabidopsis thaliana*, ACGTATERD1 was identified as a cis-acting element of the early responsive to dehydration stress 1 (erd1) gene required for early responses to dehydration stress-induced etiolation^43^. WRKY710S has been identified as a cis-acting element of the WRKY gene^44^,^45^,^46^. GT1CONSENSUS is a sequence in the promoter of pathogenesis-related protein-1a (PR-1a)^47^. The binding of GT1CONSENSUS to GT-1-like factors influences the expression levels of SA-inducible genes. The adversity and disease resistance-related elements also included the salt response element GT1GMSCAM4, defence- and stress-related element DOFCOREZM, pathogen elicitor-responsive element OSE2ROOTNODULE and mechanical injury response element WBOXNTERF3. These findings indicate that JAZ genes play key roles in resistance to stress, defence against pathogen invasion, and the vegetative and reproductive growth of the plants. The results of the present study indicate that the JAZ genes may be regulated through a variety of hormone-related transcription factors and may play an important role in hormonal regulation.

## Conclusion

In the present study, the TIFY gene family, including the JAZ genes, was identified in three cultivated species: *Gossypium hirsutum, Gossypium barbadense* and *Gossypium arboretum*. The expression patterns of the JAZ genes in the leaves of the hormone-treated upland cotton and *Verticillium dahlia*-infected sea-island cotton were analysed. This study provides information of TIFY genes in the three cultivated species and the role of cotton JAZ genes in hormonal signalling and biotic/abiotic stress resistance, and lays a foundation for further investigations of the functions of the TIFY gene family and JAZ genes in gene regulation networks of stress resistance and development.

## Materials and Methods

### Cotton and Arabidopsis materials

The cotton materials used in the studies were upland cotton variety KV-1, sea-island cotton variety Xinhai 15 and Xinhai 16.

### Identification of JAZ protein in Gossypium

The genome sequences of *G. hirsutum, G. arboreum* and *G. barbadense* were downloaded from the CottonGen database (http://cotton.cropdb.org/, http://www.cottongen.org)^35^,^36^,^37^,^49^,^50^,^51^. The TIFY domain and Jas and GATA motifs were represented in the Pfam database^52^ with (http://pfam.sanger.ac.uk) accession numbers PF06200, PF09425 and PF00320, respectively. Searches for three domains within the *G. hirsutum, G. barbadense* and *G. arboreum* databases were performed using HMMER 3.0^53^ with an E-value < 1e-6. To confirm the results obtained using the HMMER algorithm, the protein motifs were also queried against the Pfam and Smart databases.

### Sequence alignments and phylogenetic analyses

Multiple alignments of JAZ, PPD and ZML protein sequences from *Malus* × *domestica* Borkh, *Vitis vinifera, Arabidopsis thaliana* and *Oryza sativa* were performed using the ClustalW programme^54^. Phylogenetic trees were constructed with the MEGA 6.0 software^55^ using the neighbour-joining (NJ) method and a bootstrap test replicated 1000 times. Additionally, a separate phylogenetic tree was constructed with all JAZ, PPD and ZML protein sequences in cotton for further analysis. http://www.evolgenius.info/.

### Chromosomal location and gene duplication

The physical location data of *GhTIFY* and *GaTIFY* genes were retrieved from the *G. hirsutum* and *G. arboreum* genomes. The mapping of these TIFY genes was subsequently performed using MapInspect software. Gene duplication was defined according to the criteria described in previous studies: the aligned region of two sequences covers over 80% of the longer sequence, and the similarity of the aligned region is over 80%^56^,^57^. In addition, the DnaSp software was employed to calculate Ka (nonsynonymous substitution rate) and Ks (synonymous substitution rate).

### Exon/intron structure analysis and conserved motif identification

The gene structure provides important information, including disaggregated and evolutionary relationships among gene families. The JAZ, PPD and ZML genomic sequences and CDS sequences extracted from the cotton database were compared in gene structure display server programmes to determine the exon/intron organization of TIFY genes. Default parameters were used for the Multiple Em for Motif Elicitation (MEME) (http://meme-suite.org/)^58^ programme for the identification of conserved protein motifs and a maximum number of 12 motifs. The identified protein motifs were further annotated using WEBlogo (http://weblogo.berkeley.edu/)^59^.

### Analysis of cis-acting elements

According to the genome sequences of *Gossypium hirsutum* published and deposited in the Institute of Cotton Research of CAAS database (http://cgp.genomics.org.cn/), we cut out the 2000 bp of the 5′ sequence as the promoter domain of JAZ gene to analyse the cis-acting elements using the online software New Place (https://sogo.dna.affrc.go.jp).

### RNA extraction and real-time quantitative PCR

To investigate the expression of JAZ genes in upland and sea-island cotton species, RNA was extracted from the roots, stems and leaves of the upland cotton variety KV-1 and sea-island cotton variety Xinhai 15. Four weeks post-germination, the cotton seedlings were cultured in the presence of 100 μmol/L of methyl jasmonate (MeJA), 200 μmol/L of ethephon (ETH), 0.5 μmol/L of gibberellic acid (GA) and 900 μg/L of indole-3-acetic acid (IAA). The following culture conditions were used: photoperiod, 16 h light/8 h dark; humidity, 55–65%; light intensity, 1200 LX; and temperature, 25 °C. RNA was extracted from leaves collected from KV-1 cotton plants with a similar growth status. In addition, KV-1 cotton seedlings were inoculated with *Verticillium dahliae* strain V991 at a concentration of 2 × 10^5^ conidia/mL using the root dip method. The leaves were subsequently collected from the infected plants, and RNA was extracted from the collected leaves. RNA was extracted using the EASYspin Plus Plant RNA Kit (Aidlab, Beijing, China), and complementary DNA (cDNA) was synthesized via reverse transcription using the PrimeScript™ RT reagent Kit (Takara, Dalian, China). Real-time quantitative PCR was performed using a 7500 Fast Real-Time PCR System and the SYBR Green PCR master mix (SYBR^®^ Premix Ex Taq™ II (Tli RNase H Plus)). The PCR reaction comprised 5 μL of enzyme, 3 μL of cDNA product, 1 μL of primer mix containing both upstream and downstream primers, and 1 μL of DNase/RNase-free water (a total reaction volume of 10 μL). The quantitative PCR thermal cycler programme included 95 °C for 20 s, followed by a total of 40 cycles at 95 °C for 3 s and 60 °C for 30 s. All primers were synthesized at BGI (Supplemental Table 3). Ubiquitin 7 (UBQ7) was used as the reference gene.

## Additional Information

**How to cite this article**: Sun, Q. *et al*. Genome-wide identification of the TIFY gene family in three cultivated *Gossypium* species and the expression of JAZ genes. *Sci. Rep.* **7**, 42418; doi: 10.1038/srep42418 (2017).

**Publisher's note:** Springer Nature remains neutral with regard to jurisdictional claims in published maps and institutional affiliations.

## Supplementary Material

Supplementary Figures and Tables
