# Supplementary material for: Genome-wide identification of the TIFY gene family in three cultivated Gossypium species and the expression of JAZ genes
Source: Sci Rep. 2017 Feb 10;7:42418. doi: 10.1038/srep42418 (PMC5301204; doi:10.1038/srep42418)
Supplement: Supplementary Figures and Tables [file srep42418-s1.doc]

Supplementary Information

**TITLE:**

**Genome-wide identification of the TIFY gene family in** **three cultivated *Gossypium* species and the expression of JAZ genes**

**Author:**

Quan Sun^1,2*^, Guanghao Wang^1*^, Xiao Zhang^1*^, Xiangrui Zhang^1^, Peng Qiao^1^, Lu Long^1^, Youlu Yuan^3**^, Yingfan Cai^1**^

^1^State Key Laboratory of Cotton Biology, Henan Key Laboratory of Plant Stress Biology, School of Life Sciences, School of Computer and Information Engineering, Henan University, Kaifeng 475004, China.

^2^College of Bioinformation, Chongqing University of Posts and Telecommunications, Chongqing 400065, China.

^3^ State Key Laboratory of Cotton Biology, Cotton Institute of the Chinese Academy of Agricultural Sciences, Key Laboratory of Cotton Genetic Improvement, Ministry of Agriculture, Anyang, Henan 455000, China

*These authors contributed equally to this work. **Correspondence and requests for materials should be addressed to yingfancai@outlook.com，youluyuan@hotmail.com

Supplemental Figure 1


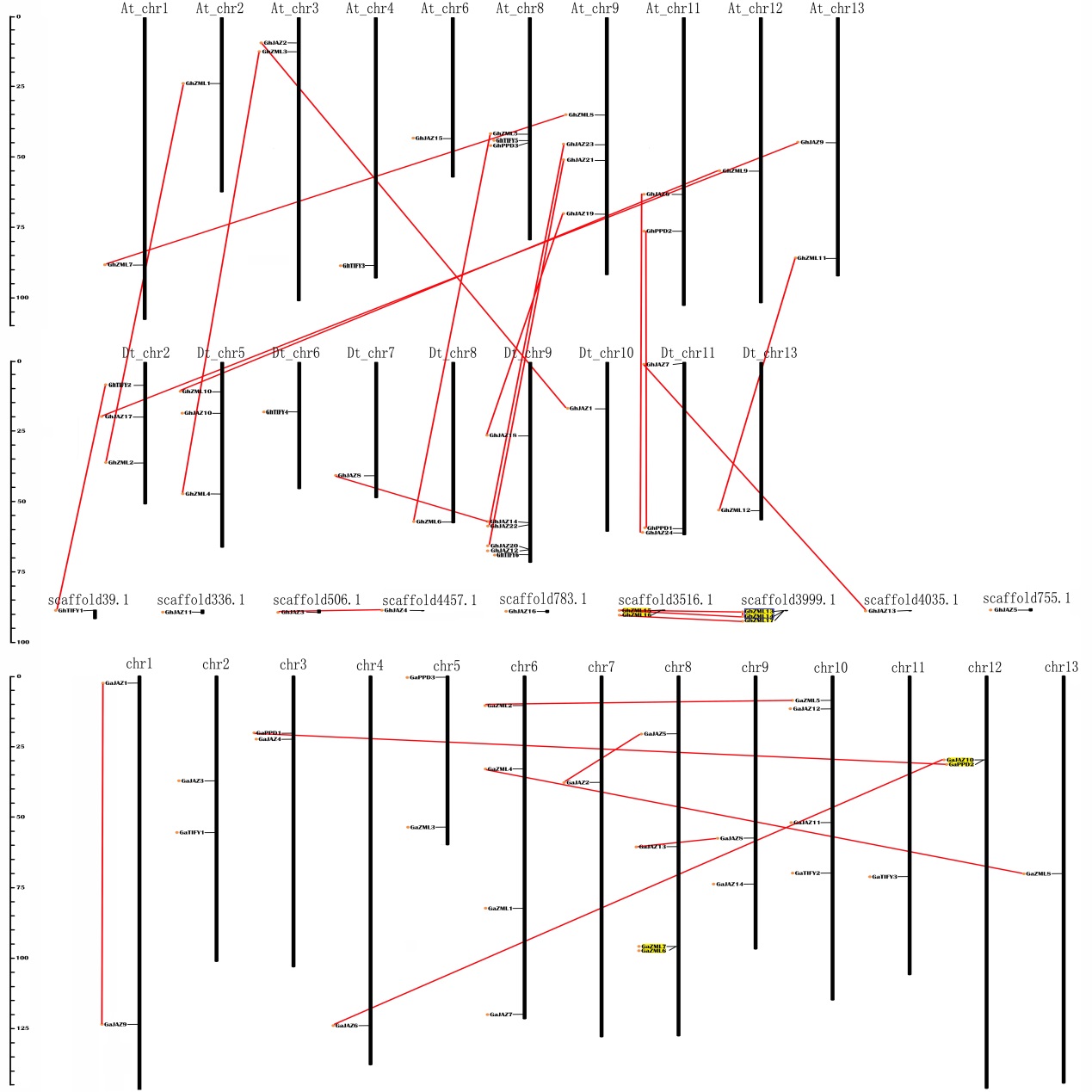


Supplemental Figure 1| Chromosomal localization of *GhTIFY* and *GaTIFY* genes. Duplication gene pairs are presented with a red line. Tandem duplicated genes highlighted with yellow box.

Supplemental Figure 2.


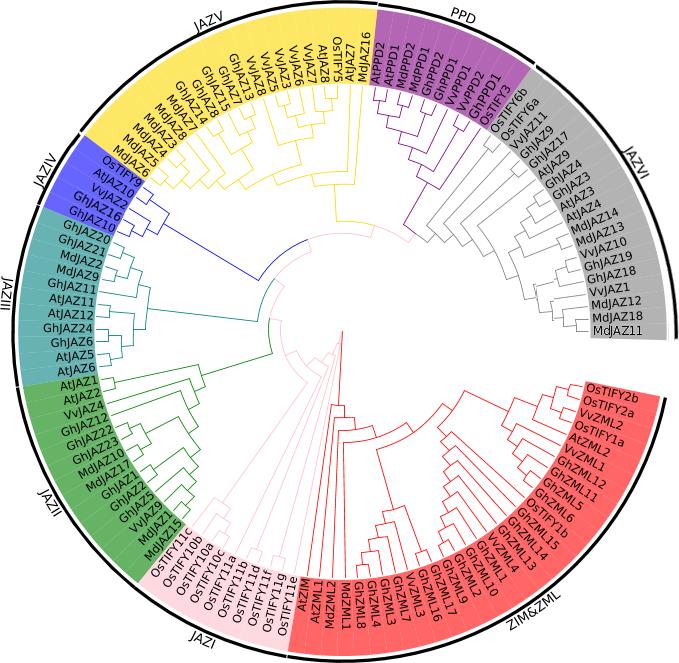


Supplemental Figure 2 | Phylogenetic analysis of JAZ, PPD and ZML genes constructed with JAZ protein sequences from *Gossypium hirsutum* L.(Gh), *Arabidopsis thaliana*(At), *Oryza sativa*(Os), *Vitis vinifera*(Vv) and *Malus* × domestica Borkh (Md).

Supplemental Figure 3.


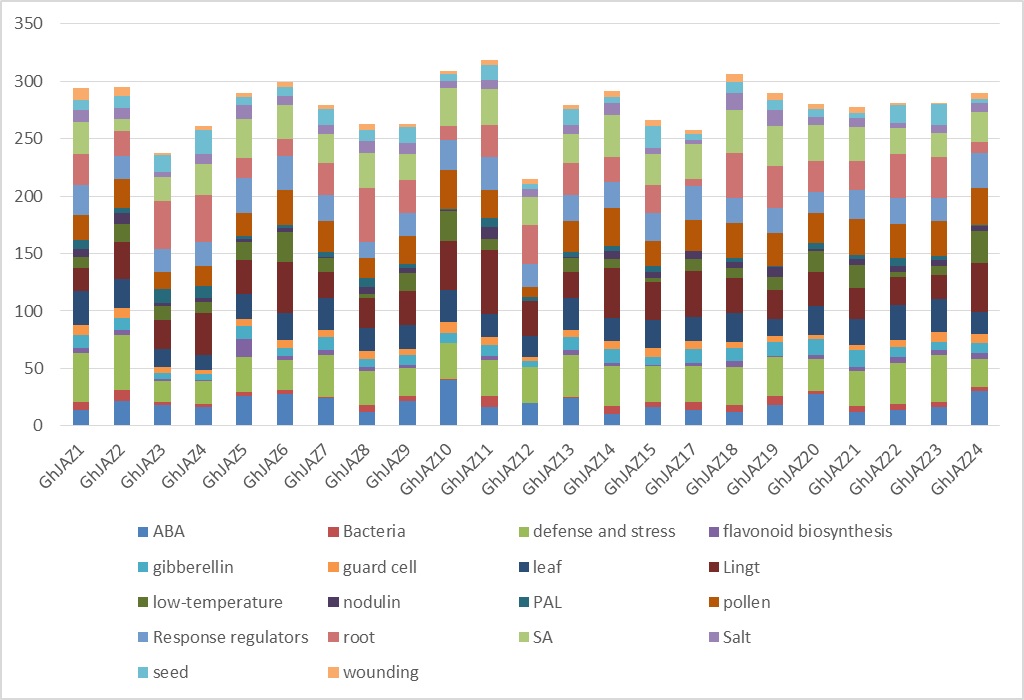
Supplementary Figure 3 | Cis-acting elements in *GhJAZ* genes.

Supplemental Table S1 | TIFY protein family in three species of cotton

| Synonym | Gene ID^A^ | Length (aa) | MW (KDa) | PI | Chr^B^ | Genome location^C^ |
| --- | --- | --- | --- | --- | --- | --- |
| GhTIFY1 | CotAD_14981 | 180 | 20.18 | 9.42 | 39.1 ^D^ | 640289-641740 (+) |
| GhTIFY2 | CotAD_49036 | 188 | 21.54 | 9.25 | Dt_chr2 | 8162459-8164057 (-) |
| GhTIFY3 | CotAD_67677 | 297 | 32.89 | 6.96 | At_chr4 | 87892869-87897808 (+) |
| GhTIFY4 | CotAD_20211 | 60 | 7.10 | 8.49 | Dt_chr6 | 17587056-17587676 (+) |
| GhTIFY5 | CotAD_12906 | 545 | 61.42 | 9.03 | At_chr8 | 43587802-43596229 (+) |
| GhTIFY6 | CotAD_50802 | 443 | 46.27 | 9.3 | Dt_chr9 | 68212202-68215824 (-) |
| GhJAZ1 | CotAD_06544 | 263 | 28.49 | 8.96 | Dt_chr10 | 16538643-16540374 (-) |
| GhJAZ2 | CotAD_46116 | 261 | 28.37 | 8.24 | At_chr3 | 8973626-8975329 (+) |
| GhJAZ3 | CotAD_21832 | 334 | 35.12 | 9.71 | 506.1 | 836874-839108 (+) |
| GhJAZ4 | CotAD_69473 | 335 | 35.30 | 9.77 | 4457.1 | 12302-14535 (+) |
| GhJAZ5 | CotAD_22999 | 223 | 24.09 | 9.14 | 755.1 | 499174-500487 (+) |
| GhJAZ6 | CotAD_62298 | 270 | 29.81 | 8.33 | At_chr11 | 62723976-62725372 (+) |
| GhJAZ7 | CotAD_26724 | 120 | 13.66 | 9.8 | Dt_chr11 | 408067-408818 (+) |
| GhJAZ8 | CotAD_27478 | 119 | 13.88 | 9.81 | Dt_chr7 | 40220954-40221863 (-) |
| GhJAZ9 | CotAD_67052 | 363 | 38.90 | 8.71 | At_chr13 | 44342298-44344785 (-) |
| GhJAZ10 | CotAD_02206 | 197 | 21.77 | 9.08 | Dt_chr5 | 18081563-18083241 (-) |
| GhJAZ11 | CotAD_18763 | 228 | 24.49 | 5.69 | 336.1 | 775854-777825 (-) |
| GhJAZ12 | CotAD_00351 | 240 | 25.54 | 9.54 | Dt_chr9 | 66433828-66435500 (-) |
| GhJAZ13 | CotAD_75527 | 120 | 13.81 | 9.72 | 4035.1 | 64676-65427 (+) |
| GhJAZ14 | CotAD_09803 | 119 | 13.71 | 9.6 | Dt_chr9 | 56839692-56840614 (+) |
| GhJAZ15 | CotAD_72619 | 125 | 14.36 | 9.87 | At_chr6 | 42892083-42893354 (+) |
| GhJAZ16 | CotAD_24822 | 242 | 26.98 | 9.64 | 783.1 | 416909-418134 (-) |
| GhJAZ17 | CotAD_52826 | 370 | 39.73 | 9.1 | Dt_chr2 | 19448038-19450540 (+) |
| GhJAZ18 | CotAD_41943 | 365 | 39.50 | 9.05 | Dt_chr9 | 26130777-26132876 (+) |
| GhJAZ19 | CotAD_63756 | 364 | 39.48 | 9.38 | At_chr9 | 69664679-69666774 (-) |
| GhJAZ20 | CotAD_00321 | 226 | 24.04 | 8.25 | Dt_chr9 | 66237218-66239656 (-) |
| GhJAZ21 | CotAD_40859 | 216 | 23.15 | 8.25 | At_chr9 | 50629016-50631468 (-) |
| GhJAZ22 | CotAD_09712 | 226 | 24.88 | 8.44 | Dt_chr9 | 57682113-57683351 (+) |
| GhJAZ23 | CotAD_26962 | 226 | 24.99 | 9.15 | At_chr9 | 45048475-45049713 (+) |
| GhJAZ24 | CotAD_21952 | 270 | 29.79 | 8.33 | Dt_chr11 | 60565219-60566607 (-) |
| GhPPD1 | CotAD_76249 | 365 | 40.30 | 6.77 | Dt_chr11 | 58962078-58966618 (-) |
| GhPPD2 | CotAD_75535 | 428 | 47.51 | 6.19 | At_chr11 | 75766620-75771286 (-) |
| GhPPD3 | CotAD_44751 | 337 | 37.30 | 6.37 | At_chr8 | 44330607-44335238 (-) |
| GhZML1 | CotAD_55806 | 356 | 38.76 | 5.04 | At_chr2 | 23402338-23406441 (-) |
| GhZML2 | CotAD_19553 | 359 | 38.83 | 4.92 | Dt_chr2 | 35736685-35740677 (-) |
| GhZML3 | CotAD_53093 | 263 | 28.54 | 8.94 | At_chr3 | 12081749-12085970 (+) |
| GhZML4 | CotAD_32387 | 269 | 29.14 | 6.99 | Dt_chr5 | 46673797-46677397 (-) |
| GhZML5 | CotAD_35015 | 286 | 30.67 | 5.4 | At_chr8 | 41364175-41366379 (-) |
| GhZML6 | CotAD_50979 | 300 | 31.93 | 5.28 | Dt_chr8 | 56644469-56646761 (+) |
| GhZML7 | CotAD_30081 | 282 | 30.66 | 6.5 | At_chr1 | 87727338-87731461 (-) |
| GhZML8 | CotAD_63029 | 262 | 28.60 | 8.18 | At_chr9 | 34522807-34525751 (+) |
| GhZML9 | CotAD_36871 | 348 | 38.63 | 5.52 | At_chr12 | 54406400-54411497 (-) |
| GhZML10 | CotAD_37669 | 348 | 38.57 | 5.41 | Dt_chr5 | 10474674-10479301 (-) |
| GhZML11 | CotAD_65649 | 322 | 33.88 | 5.57 | At_chr13 | 85359078-85362246 (+) |
| GhZML12 | CotAD_47238 | 286 | 30.50 | 5.83 | Dt_chr13 | 52595353-52598230 (+) |
| GhZML13 | CotAD_67270 | 318 | 34.66 | 5.01 | 3999.1 | 22707-26567 (-) |
| GhZML14 | CotAD_67271 | 353 | 38.50 | 4.83 | 3999.1 | 30474-34451 (-) |
| GhZML15 | CotAD_68173 | 353 | 38.50 | 4.83 | 3516.1 | 40290-44267 (-) |
| GhZML16 | CotAD_68174 | 287 | 30.94 | 5.48 | 3516.1 | 50446-54149 (-) |
| GhZML17 | CotAD_67272 | 296 | 31.80 | 5.46 | 3999.1 | 40610-44832 (-) |
| GbTIFY1 | Gbscaffold102.27 | 348 | 38.75 | 8.42 | D08 | 10235486-10240554 (+) |
| GbTIFY2 | Gbscaffold10257.4 | 160 | 18.15 | 9.52 | D01 | 55523424-55525029 (-) |
| GbTIFY3 | Gbscaffold11436.2 | 151 | 17.23 | 8.3 | 11436_D05 | 11017-12875 (+) |
| GbTIFY4 | Gbscaffold3656.12 | 488 | 54.35 | 7.84 | D12 | 467605-475378 (+) |
| GbTIFY5 | Gbscaffold5097.4 | 429 | 44.61 | 9.32 | 5097 | 43980-48095 (+) |
| GbJAZ01 | Gbscaffold955.7 | 263 | 28.48 | 9.24 | D06 | 13957804-13962738 (+) |
| GbJAZ02 | Gbscaffold8688.15 | 240 | 25.56 | 9.21 | A05 | 1616934-1619111 (-) |
| GbJAZ03 | Gbscaffold2620.6 | 263 | 28.62 | 8.24 | A06 | 19079989-19082725 (+) |
| GbJAZ04* |  | 335 | 86.36 | 5.02 |  |  |
| GbJAZ05 | Gbscaffold19048.28 | 234 | 25.25 | 9.04 | D08 | 68794295-68796158 (-) |
| GbJAZ06 | Gbscaffold687.3 | 270 | 29.81 | 8.33 | 687 | 27347-29247 (-) |
| GbJAZ07 | Gbscaffold9879.1 | 120 | 13.66 | 9.8 | D10 | 3918649-3920897 (+) |
| GbJAZ08 | Gbscaffold2710.2 | 119 | 13.88 | 9.81 | A05 | 10802886-10804280 (-) |
| GbJAZ09 | Gbscaffold24022.2 | 362 | 38.85 | 9 | D01 | 41824831-41827929 (-) |
| GbJAZ10 | Gbscaffold2510.10 | 197 | 21.82 | 9.34 | A03 | 93428129-93430477 (+) |
| GbJAZ11 | Gbscaffold26079.19 | 228 | 24.40 | 5.96 | D07 | 1841956-1844553 (+) |
| GbJAZ12 | Gbscaffold6720.14 | 240 | 25.54 | 9.54 | D04 | 29668396-29671130 (-) |
| GbJAZ13 | Gbscaffold10262.6 | 228 | 24.49 | 5.69 | 10262_A07 | 98599-101177 (+) |
| GbJAZ14 | Gbscaffold4563.1 | 516 | 47.25 | 9.42 | A01 | 75472374-75475396 (+) |
| GbJAZ15 | Gbscaffold9188.5 | 125 | 14.35 | 10.12 | D09 | 34273210-34275030 (+) |
| GbJAZ16 | Gbscaffold15070.5 | 190 | 21.21 | 9.89 | A01 | 1141987-1145401 (-) |
| GbJAZ17 | Gbscaffold5037.1 | 363 | 38.90 | 8.71 | 5037 | 11893-15137 (-) |
| GbJAZ18 | Gbscaffold197.3 | 364 | 39.48 | 9.38 | A05 | 43101520-43103955 (-) |
| GbJAZ19 | Gbscaffold2024.14 | 365 | 39.47 | 9.05 | D05 | 39281965-39284591 (-) |
| GbJAZ20 | Gbscaffold3667.18 | 229 | 24.41 | 8.25 | A05 | 68134401-68137663 (+) |
| GbJAZ21 | Gbscaffold10000.41 | 226 | 24.04 | 8.25 | D05 | 3317879-3321053 (-) |
| GbJAZ22 | Gbscaffold19167.8 | 369 | 40.97 | 9.49 | D05 | 12729745-12735154 (+) |
| GbJAZ23 | Gbscaffold26652.3 | 226 | 24.89 | 8.98 | 26652 | 24081-26397 (+) |
| GbJAZ24 | Gbscaffold13982.7 | 247 | 27.69 | 9.08 | D10 | 5311379-5312662 (-) |
| GbJAZ25 | Gbscaffold21128.45 | 234 | 25.26 | 8.89 | A08 | 108210244-108212087 (+) |
| GbJAZ26 | Gbscaffold13138.23 | 190 | 21.18 | 10.04 | D01 | 1952286-1955656 (+) |
| GbJAZ27 | Gbscaffold358.7 | 125 | 14.34 | 9.87 | A09 | 54281656-54283282 (-) |
| GbJAZ28 | Gbscaffold13982.6 | 270 | 29.79 | 8.33 | D10 | 5300116-5302090 (-) |
| GbJAZ29 | Gbscaffold8688.13 | 153 | 16.44 | 9.99 | A05 | 1612133-1613204 (-) |
| GbPPD1 | Gbscaffold4531.1 | 365 | 40.30 | 6.77 | D10 | 380777-38083312 (+) |
| GbPPD2 | Gbscaffold2186.1 | 366 | 40.41 | 7.09 | 2186_A10 | 135353-140790 (+) |
| GbPPD3 | Gbscaffold673.20 | 339 | 37.49 | 6.37 | A12 | 351356-356786 (-) |
| GbPPD4 | Gbscaffold8008.5 | 344 | 38.42 | 9.24 | A08 | 10505406-10510842 (-) |
| GbZML01 | Gbscaffold14105.2 | 314 | 33.41 | 5.41 | A12 | 2406887-2408090 (-) |
| GbZML02 | Gbscaffold17078.7 | 348 | 38.61 | 5.41 | D02 | 66827909-66833162 (+) |
| GbZML03 | Gbscaffold9862.2 | 382 | 41.75 | 4.9 | A01 | 36111824-36116905 (+) |
| GbZML04 | Gbscaffold4651.27 | 337 | 36.59 | 7.56 | D05 | 26601376-26608068 (+) |
| GbZML05 | Gbscaffold11033.7 | 348 | 38.57 | 5.52 | A03 | 98103231-98109902 (+) |
| GbZML06 | Gbscaffold14016.5 | 314 | 33.26 | 5.19 | D12 | 2273876-2276680 (+) |
| GbZML07 | Gbscaffold1009.2 | 282 | 30.66 | 7.1 | A05 | 24647855-24652629 (-) |
| GbZML08 | Gbscaffold698.4 | 285 | 30.68 | 6.07 | D02 | 14940709-14944710 (-) |
| GbZML09 | Gbscaffold1520.23 | 314 | 33.31 | 5.83 | 1520 | 433428-437524 (+) |
| GbZML10 | Gbscaffold16457.3 | 312 | 33.42 | 5.57 | A13 | 6479063-6483246 (-) |
| GbZML11 | Gbscaffold2267.7 | 329 | 35.53 | 4.98 | D01 | 23666188-23670541 (+) |
| GbZML12 | Gbscaffold26361.6 | 352 | 38.39 | 4.78 | D06 | 2624960-2629935 (+) |
| GbZML13 | Gbscaffold498.10 | 353 | 38.50 | 4.83 | A06 | 3267760-3272785 (+) |
| GbZML14 | Gbscaffold26361.5 | 304 | 28.62 | 8.24 | D06 | 2601743-2607773 (+) |
| GbZML15 | Gbscaffold498.11 | 287 | 30.94 | 5.48 | A06 | 3280395-3284743 (-) |
| GbZML16 | Gbscaffold14016.4 | 273 | 29.28 | 6.6 | D12 | 2270480-2272062 (+) |
| GaTIFY1 | Cotton_A_27045 | 180 | 20.18 | 9.42 | chr2 | 55430055-55431505 (-) |
| GaTIFY2 | Cotton_A_11516 | 427 | 44.44 | 9.32 | chr10 | 69750629-69754033 (+) |
| GaTIFY3 | Cotton_A_23690 | 66 | 7.83 | 7.77 | chr11 | 71031290-71031713 (-) |
| GaJAZ01 | Cotton_A_05658 | 228 | 24.52 | 5.69 | chr1 | 2454140-2456131 (+) |
| GaJAZ02 | Cotton_A_00049 | 243 | 27.13 | 9.51 | chr7 | 37588035-37589269 (-) |
| GaJAZ03 | Cotton_A_36376 | 363 | 38.84 | 8.69 | chr2 | 37063169-37065656 (+) |
| GaJAZ04 | Cotton_A_11862 | 252 | 27.34 | 8.89 | chr3 | 22379114-22380520 (-) |
| GaJAZ05 | Cotton_A_10012 | 197 | 21.87 | 9.43 | chr8 | 20431252-20432955 (-) |
| GaJAZ06 | Cotton_A_18896 | 270 | 29.85 | 8.64 | chr4 | 123852070-123853467 (-) |
| GaJAZ07 | Cotton_A_01448 | 1114 | 122.00 | 9.42 | chr6 | 119880121-119892245 (-) |
| GaJAZ08 | Cotton_A_27840 | 240 | 25.61 | 9.06 | chr9 | 57404839-57406509 (-) |
| GaJAZ09 | Cotton_A_14056 | 229 | 24.40 | 8.25 | chr1 | 123420039-123422497 (+) |
| GaJAZ10 | Cotton_A_02654 | 244 | 27.07 | 9.33 | chr12 | 2970615-2972402 (-) |
| GaJAZ11 | Cotton_A_12336 | 119 | 13.88 | 9.55 | chr10 | 51819759-51820669 (-) |
| GaJAZ12 | Cotton_A_36075 | 364 | 39.47 | 9.38 | chr10 | 11517075-11519170 (+) |
| GaJAZ13 | Cotton_A_09418 | 261 | 28.35 | 8.24 | chr8 | 60402392-60404087 (-) |
| GaJAZ14 | Cotton_A_02904 | 120 | 13.77 | 9.5 | chr9 | 73579784-73580535 (-) |
| GaPPD1 | Cotton_A_16991 | 359 | 39.81 | 8.52 | chr3 | 20197463-20201830 (+) |
| GaPPD2 | Cotton_A_22024 | 339 | 37.54 | 6.83 | chr12 | 2974352-2978986 (-) |
| GaPPD3 | Cotton_A_41299 | 415 | 46.21 | 6.05 | chr5 | 286257-290962 (+) |
| GaZML1 | Cotton_A_28500 | 356 | 38.86 | 5.05 | chr6 | 82260117-82264184 (+) |
| GaZML2 | Cotton_A_30531 | 288 | 31.05 | 6.38 | chr6 | 1033534-1037866 (-) |
| GaZML3 | Cotton_A_20830 | 363 | 40.32 | 5.69 | chr5 | 53452604-53457246 (+) |
| GaZML4 | Cotton_A_15821 | 314 | 33.37 | 5.29 | chr6 | 32800822-32803007 (-) |
| GaZML5 | Cotton_A_16692 | 261 | 28.55 | 8.47 | chr10 | 8553629-8557974 (-) |
| GaZML6 | Cotton_A_24824 | 353 | 38.50 | 4.83 | chr8 | 95097516-95101493 (+) |
| GaZML7 | Cotton_A_24823 | 296 | 31.81 | 5.46 | chr8 | 95087719-95091450 (+) |
| GaZML8 | Cotton_A_17214 | 312 | 33.42 | 5.57 | chr13 | 69883347-69886390 (+) |

^A^ :Accession numbers of detabase locus ID. ^B^ :”Dt” means gene located of D genome.“At” means gene located A genome. ^C^ :Located site of the TIFY genes on chromosome. ^D^: all the serial number mean the abbreviation of scaffold, such as “scaffold39.1” to “39.1”. * :Cloned in sea-island cotton.

Supplemental Table S2| Synonymous and nonsynonymous substitution rates and the estimated dates for the duplication events in TIFY genes of cotton

| Gene1 | Gene2 | Ka | Ks | Ka/Ks |
| --- | --- | --- | --- | --- |
| GbJAZ02 | GbJAZ12 | 0.015 | 0.024 | 0.615 |
| GbJAZ03 | GbJAZ01 | 0.023 | 0.028 | 0.836 |
| GbJAZ13 | GbJAZ11 | 0.022 | 0.049 | 0.447 |
| GbJAZ16 | GbJAZ26 | 0.007 | 0.038 | 0.183 |
| GbJAZ18 | GbJAZ19 | 0.021 | 0.024 | 0.877 |
| GbJAZ20 | GbJAZ21 | 0.014 | 0.049 | 0.287 |
| GbJAZ25 | GbJAZ05 | 0.011 | 0.049 | 0.231 |
| GbJAZ27 | GbJAZ15 | 0.021 | 0.048 | 0.441 |
| GbJAZ29 | GbJAZ12 | 0 | 0 | 0 |
| GbZML01 | GbZML06 | 0.014 | 0.05 | 0.283 |
| GbZML03 | GbZML11 | 0.04 | 0.035 | 1.145 |
| GbZML05 | GbZML02 | 0.008 | 0.03 | 0.253 |
| GbZML07 | GbZML04 | 0.005 | 0.005 | 0.94 |
| GbZML13 | GbZML12 | 0.007 | 0.025 | 0.296 |
| GbZML15 | GbZML14 | 0.091 | 0.152 | 0.597 |
| GbPPD2 | GbPPD1 | 0.013 | 0.05 | 0.263 |
| GbPPD4 | GbTIFY1 | 0.016 | 0.029 | 0.545 |
| GaJAZ01 | GbJAZ13 | 0.002 | 0 | 0 |
| GaJAZ02 | GbJAZ10 | 0.019 | 0.046 | 0.406 |
| GaJAZ04 | GbJAZ25 | 0.006 | 0.018 | 0.311 |
| GaJAZ08 | GbJAZ02 | 0.007 | 0.018 | 0.408 |
| GaJAZ09 | GbJAZ20 | 0.002 | 0.006 | 0.328 |
| GaJAZ11 | GbJAZ08 | 0.004 | 0.038 | 0.095 |
| GaJAZ12 | GbJAZ18 | 0.004 | 0.008 | 0.462 |
| GaJAZ13 | GbJAZ03 | 0.003 | 0.011 | 0.297 |
| GaZML1 | GbZML03 | 0.005 | 0.012 | 0.402 |
| GaZML3 | GbZML05 | 0.003 | 0.009 | 0.294 |
| GaZML4 | GbZML01 | 0.006 | 0.009 | 0.636 |
| GaZML5 | GbZML07 | 0.009 | 0.01 | 0.926 |
| GaZML6 | GbZML13 | 0 | 0 | 0 |
| GaZML7 | GbZML15 | 0.003 | 0.01 | 0.32 |
| GaZML8 | GbZML10 | 0 | 0 | 0 |
| GaPPD1 | GbPPD4 | 0 | 0.013 | 0 |
| GaPPD2 | GbPPD3 | 0.013 | 0.018 | 0.715 |
| GaPPD3 | GbPPD2 | 0.011 | 0.013 | 0.881 |
| GrJAZ01 | GbJAZ11 | 0.002 | 0.012 | 0.16 |
| GrJAZ02 | GbJAZ26 | 0.002 | 0.022 | 0.103 |
| GrJAZ03 | GbJAZ09 | 0.007 | 0.012 | 0.605 |
| GrJAZ04 | GbJAZ05 | 0.008 | 0.052 | 0.153 |
| GrJAZ06 | GbJAZ15 | 0 | 0.023 | 0 |
| GrJAZ08 | GbJAZ12 | 0.006 | 0 | 0 |
| GrJAZ09 | GbJAZ21 | 0.002 | 0.012 | 0.169 |
| GrJAZ10 | GbJAZ22 | 0.002 | 0.014 | 0.143 |
| GrJAZ12 | GbJAZ19 | 0.01 | 0.024 | 0.409 |
| GrJAZ13 | GbJAZ1 | 0.004 | 0.013 | 0.305 |
| GrJAZ14 | GbJAZ07 | 0.004 | 0.012 | 0.303 |
| GrJAZ15 | GbJAZ28 | 0.002 | 0.011 | 0.142 |
| GrZML1 | GbZML11 | 0.042 | 0.044 | 0.948 |
| GrZML3 | GbZML02 | 0.004 | 0.021 | 0.175 |
| GrZML4 | GbZML06 | 0.003 | 0.009 | 0.318 |
| GrZML5 | GbZML04 | 0.005 | 0.03 | 0.155 |
| GrZML6 | GbZML12 | 0.006 | 0.008 | 0.756 |
| GrZML7 | GbZML14 | 0.109 | 0.132 | 0.825 |
| GrZML8 | GbZML06 | 0.092 | 0.395 | 0.232 |
| GrTIFY1 | GbTIFY2 | 0.091 | 0.11 | 0.823 |
| GrPPD1 | GbTIFY1 | 0.016 | 0.018 | 0.862 |
| GrPPD2 | GbTIFY4 | 0.013 | 0.021 | 0.618 |
| GrPPD3 | GbPPD1 | 0.01 | 0.016 | 0.624 |
| GhJAZ2 | GhJAZ1 | 0.02 | 0.022 | 0.902 |
| GhJAZ6 | GhJAZ24 | 0.018 | 0.046 | 0.381 |
| GhJAZ9 | GhJAZ17 | 0.021 | 0.028 | 0.731 |
| GhJAZ19 | GhJAZ18 | 0.019 | 0.024 | 0.822 |
| GhJAZ21 | GhJAZ20 | 0.008 | 0.046 | 0.185 |
| GhJAZ23 | GhJAZ22 | 0.023 | 0.056 | 0.416 |
| GhPPD2 | GhPPD1 | 0.018 | 0.059 | 0.306 |
| GhZML1 | GhZML2 | 0.047 | 0.053 | 0.895 |
| GhZML3 | GhZML4 | 0.051 | 0.072 | 0.713 |
| GhZML5 | GhZML6 | 0.012 | 0.056 | 0.219 |
| GhZML9 | GhZML10 | 0.008 | 0.03 | 0.253 |
| GhZML11 | GhZML12 | 0.036 | 0.099 | 0.359 |
| GaJAZ3 | GhJAZ9 | 0.007 | 0.004 | 1.8 |
| GaJAZ6 | GhJAZ6 | 0.005 | 0 | 0 |
| GaJAZ12 | GhJAZ19 | 0.004 | 0.004 | 0.923 |
| GaJAZ13 | GhJAZ2 | 0.003 | 0.011 | 0.297 |
| GaPPD1 | GhTIFY3 | 0.053 | 0.08 | 0.665 |
| GaPPD2 | GhPPD3 | 0.003 | 0.008 | 0.31 |
| GaPPD3 | GhPPD2 | 0.005 | 0.004 | 1.444 |
| GaZML1 | GhZML1 | 0.006 | 0.008 | 0.753 |
| GaZML2 | GhZML3 | 0.004 | 0.006 | 0.603 |
| GaZML3 | GhZML9 | 0.003 | 0.004 | 0.595 |
| GaZML4 | GhZML5 | 0.009 | 0.01 | 0.929 |
| GaZML5 | GhZML8 | 0.064 | 0.053 | 1.206 |
| GaZML5 | GhZML7 | 0.014 | 0.02 | 0.686 |
| GaZML8 | GhZML11 | 0.001 | 0 | 0 |
| GrJAZ03 | GhJAZ17 | 0.007 | 0.012 | 0.605 |
| GrJAZ05 | GhJAZ10 | 0.038 | 0.032 | 1.167 |
| GrJAZ08 | GhJAZ12 | 0.006 | 0 | 0 |
| GrJAZ09 | GhJAZ20 | 0.002 | 0.006 | 0.339 |
| GrJAZ10 | GhJAZ22 | 0 | 0 | 0 |
| GrJAZ11 | GhJAZ14 | 0.011 | 0.012 | 0.902 |
| GrJAZ12 | GhJAZ18 | 0.008 | 0.02 | 0.431 |
| GrJAZ13 | GhJAZ1 | 0.002 | 0.007 | 0.303 |
| GrJAZ14 | GhJAZ7 | 0.004 | 0.036 | 0.1 |
| GrJAZ15 | GhJAZ24 | 0.002 | 0.011 | 0.142 |
| GrZML1 | GhZML2 | 0.012 | 0.01 | 1.25 |
| GrZML2 | GhZML4 | 0.031 | 0.062 | 0.51 |
| GrZML3 | GhZML10 | 0.005 | 0.017 | 0.296 |
| GrZML4 | GhZML6 | 0.003 | 0.005 | 0.63 |
| GrZML6 | GhZML2 | 0.109 | 0.316 | 0.345 |
| GrZML8 | GhZML12 | 0.029 | 0.059 | 0.487 |
| GrTIFY2 | GhTIFY6 | 0.01 | 0.008 | 1.266 |
| GrPPD3 | GhPPD1 | 0.01 | 0.012 | 0.852 |

Orthologous or paralogs gene sets between the A genome, D genome and four subgenomes of *Gossypium hirsutum*(AtDt), *Gossypium barbadense*(At'Dt'), *Gossypium* *arboreum*(A) and *Gossypium raimondii*(D).

Supplemental Table S3 | The primers for RT-qPCR

| Gene name1 | Gene name2 | Forward primer | Reverse primer |
| --- | --- | --- | --- |
| GhJAZ1/2 | GbJAZ1/3 | CGGTATCGGGGTTCCTCCTA | TTGGTAGATCGCCGGGAATG |
| GhJAZ3/4 | GbJAZ4 | AAAGCCACGCATGACACTCT | CGCATAGTGGTTTCCCCCTT |
| GhJAZ5 | GbJAZ5/25 | TGAATGCGTTCGTCCCA | CGGAGCCGTTTATTGG |
| GhJAZ6/24 | GbJAZ6/24 | TGGTGTTGTTGCCGACTCTT | ACGGTTGCAGAGGTGATGTT |
| GhJAZ7/13 | GbJAZ7 | CTGCAACTTGGAGCTTCGTC | TCGTTCATCCGTTTCTCGGT |
| GhJAZ8/14 | GbJAZ8/14 | AACCCAGCAGCAACAGCTAA | ATGGCTTTTGCCTGAAGCTC |
| GhJAZ9 | GbJAZ9 | GCCTTCTGTTCCACAGGCTC | TGCATTCGATTCCATGGTAGTT |
| GhJAZ10 | GbJAZ10 | CCCGCAGGGTCAGGATAATG | GGGAGCTGTTTCAGGACCAT |
| GhJAZ11 | GbJAZ11/13 | AATCCCAACTCACCATCT | GCCACAGTAGCAGCAATA |
| GhJAZ12 | GbJAZ12 | GAGAGCTTCACTGCATCGGT | GAGCCATGACTTGTTGTCGC |
| GhJAZ15 | GbJAZ15 | TACAGAGCTTCAGGCAAGAGC | GAATCCGGTTCCGATCCTGT |
| GhJAZ17 | GbJAZ17 | TGCCTTCTGGTATCCACCCT | TTGCGCTTCTCCAAAAACCG |
| GhJAZ18/19 | GbJAZ18/19 | CCGGTCTCGAACCAATCAGT | TTCCACCAAAGGAACCGAGG |
| GhJAZ20 | GbJAZ20 | GCAACTCCTGCATCTGGACTG | GTGAGGTGGTGGCACAATCA |
| GhJAZ21 | GbJAZ21 | GAACTGCCCTGCTCCATCTCAA | CAGCAGTTGCGGCAATAAGC |
| GhJAZ22/23 | GbJAZ22/23 | TAAAACCGAATCTCGGGGCA | ACTGCCACTTGACTGCCAAA |
| GhUB7 | GbUB7 | AAGGCATTCCACCTGACCAAC | CTTGACCTTCTTCTTCTTGTGCTTG |
